# Protective Effect of *EBF Transcription Factor 1* (*EBF1*) Polymorphism in Sporadic and Familial Spontaneous Preterm Birth: Insights from a Case-Control Study

**DOI:** 10.3390/ijms252011192

**Published:** 2024-10-17

**Authors:** Tea Mladenić, Jasenka Wagner, Mirta Kadivnik, Nina Pereza, Saša Ostojić, Borut Peterlin, Sanja Dević Pavlić

**Affiliations:** 1 Department of Medical Biology and Genetics, Faculty of Medicine, University of Rijeka, 51000 Rijeka, Croatia; tea.mladenic@uniri.hr (T.M.); nina.pereza@uniri.hr (N.P.); sasa.ostojic@uniri.hr (S.O.); sanja.devic@uniri.hr (S.D.P.); 2 Department of Medical Biology and Genetics, Faculty of Medicine, Josip Juraj Strossmayer University, 31000 Osijek, Croatia; jwagner@mefos.hr; 3 Department of Obstetrics and Gynecology, Faculty of Medicine, Josip Juraj Strossmayer University, 31000 Osijek, Croatia; mirta.kadivnik@gmail.com; 4 Clinic of Obstetrics and Gynecology, University Hospital Center Osijek, 31000 Osijek, Croatia; 5 Clinical Institute of Medical Genetics, University Medical Center Ljubljana, 1000 Ljubljana, Slovenia

**Keywords:** preterm birth, single-nucleotide polymorphism, gene, genetic association studies

## Abstract

This study investigated the potential role of specific single-nucleotide polymorphisms (SNPs) in the genes *Astrotactin 1* (*ASTN1*), *EBF Transcription Factor 1* (*EBF1*), *Eukaryotic Elongation Factor*, *Selenocysteine-tRNA Specific* (*EEFSEC*), *Microtubule-Associated Serine/Threonine Kinase 1* (*MAST1*), and *Tumor Necrosis Factor Alpha* (*TNF-α*) to assess whether these genetic variants contribute to the risk of spontaneous preterm birth (sPTB). A case-control study was conducted involving 573 women from Croatia and Slovenia: 248 with sporadic sPTB (positive personal and negative family history of sPTB before 37 weeks’ gestation), 44 with familial sPTB (positive personal and family history of sPTB before 37 weeks’ gestation), and 281 control women. The analysis of *ASTN1* rs146756455, *EBF1* rs2963463, *EBF1* rs2946169, *EEFSEC* rs201450565, *MAST1* rs188343966, and *TNF-α* rs1800629 SNPs was performed using TaqMan real-time PCR. *p*-values were Bonferroni-adjusted for multiple comparisons. *EBF1* SNP rs2963463 was significantly associated with sPTB (*p* adj = 0.03). Women carrying the CC genotype had a 3–4-times lower risk of sPTB (*p* adj < 0.0001). In addition, a significant difference in the frequency of the minor C allele was observed when comparing familial sPTB cases with controls (*p* adj < 0.0001). All other associations were based on unadjusted *p*-values. The minor T allele of *EBF1* SNP rs2946169 was more frequent in sPTB cases overall than in controls, especially in sporadic sPTB (*p* = 0.045). Similarly, the CC genotype of *ASTN1* SNP rs146756455 was more frequent in sporadic sPTB cases compared to controls (*p* = 0.019). Finally, the *TNF-α* SNP rs1800629 minor A allele and AA genotype were more common in the familial sPTB group compared to sporadic sPTB and controls (*p* < 0.05). The *EBF1* SNP rs2963463 polymorphism showed a protective effect in the pathogenesis of sPTB, particularly in women carrying the CC genotype. Moreover, *EBF1* SNP rs2946169 and *ASTN1* SNP rs146756455, as well as *TNF-α* SNP rs1800629, were associated with an increased risk of sPTB, representing suggestive potential risk factors for sporadic and familial sPTB, respectively.

## 1. Introduction

Preterm birth (PTB), defined as birth occurring before the 37th week of gestation, impacts 6.9% of pregnancies in the European population [1]. It is the leading cause of infant mortality and morbidity, associated with a range of health complications such as underdeveloped lungs, kidneys, brain, and cardiovascular system, potentially affecting organ structure and metabolism, and increasing the risk of chronic disease later in life [2]. Despite significant research efforts and advancements in healthcare, the prevention of preterm birth remains a challenge, largely due to the incomplete understanding of its underlying causes.

Preterm births are classified as iatrogenic, medically indicated, or spontaneous. While iatrogenic and medically indicated preterm births are associated with specific maternal or fetal complications, the etiology of spontaneous preterm birth (sPTB), characterized by spontaneous onset labor with or without premature rupture of membranes (PPROM) [3], is predominantly idiopathic. However, the recurrence of sPTB in mothers and daughters, and between sisters, suggests that genetic factors may play a crucial role in transmission within the family [4,5].

Research indicates that maternal genetic variants contribute around 20.6 to 25% to the heritability of sPTB [6,7]. A strong genetic predisposition is suggested by a positive family history, particularly when sPTB occurs on the maternal side, such as in mothers or sisters, as evidenced by higher recurrence rates in these cases. These familial patterns emphasize the necessity of identifying maternal genetic variants to improve understanding of the mechanisms leading to sPTB.

Among the various genetic targets studied to identify specific risk factors for sPTB, no consensus was reached regarding the most significant associations. In our recent comprehensive systematic review and meta-analysis of all studies published between 1999 and 2023 that investigated the genetic association between the maternal genome and the occurrence of sPTB, we identified six potentially critical single-nucleotide polymorphisms (SNPs) in the following genes: *Astrotactin 1* (*ASTN1*), *EBF Transcription Factor 1* (*EBF1*), *Eukaryotic Elongation Factor*, *Selenocysteine-tRNA Specific* (*EEFSEC*), *Microtubule-Associated Serine*/*Threonine Kinase 1* (*MAST1*), and *Tumor Necrosis Factor Alpha* (*TNF-α*) [8].

The SNP from the *TNF-α* gene was chosen as a candidate because, after conducting a meta-analysis of candidate gene studies, it was the only one found to be significant in association with sPTB [8]. Given that the production of proinflammatory cytokines, such as *TNF-α*, contributes to the initiation of labor by promoting the infiltration of inflammatory cells into the cervix, myometrium, chorioamniotic membrane, and amniotic cavity, certain SNPs may prematurely trigger this inflammatory response, potentially leading to preterm labor [9].

From the largest maternal genome-wide association study (GWAS) to date, three SNPs in *EBF1* and *EEFSEC* showed global significance in relation to sPTB. SNPs in *EEFSEC* may impact selenoprotein synthesis, crucial for antioxidant defenses, thyroid regulation, placental health, and immune function during pregnancy [10]. In contrast, SNPs in *EBF1*, essential for B-cell development, have been linked to reduced mRNA levels in the second and/or third trimesters in women with sPTB, increasing the odds of sPTB by 2.9 to 4.3 times [11].

A more recent GWAS by Gupta et al., investigating different sPTB phenotypes, identified two significant SNPs in the *ASTN1* and *MAST1* genes [12]. Both *MAST1* and *ASTN1* are involved in neural development and migration. While their direct association with the initiation of PTB is unclear, variations in the *MAST1* gene have been linked to mega-corpus callosum syndrome, which is characterized by cerebellar hypoplasia and cortical abnormalities [13].

In this study, we aim to elucidate the possible associations between polymorphisms in the candidate genes *ASTN1*, *EBF1*, *EEFSEC*, *MAST1*, and *TNF-α* and the occurrence of sPTB. By focusing on a cohort with a positive family history for sPTB, we seek to identify the most significant genetic associations. Additionally, we aim to explore the relationship between these candidate SNPs and various clinical characteristics in women with sPTB and their newborns. Through this comprehensive approach, we will clarify the impact of both genetic and clinical factors on the incidence of sPTB.

## 2. Results

The clinical characteristics of the women with familial and sporadic sPTB and the control women and their newborns are shown in Table 1.

There was a statistically significant difference in parity: women with familial sPTB had more singleton pregnancies than those with sporadic sPTB and the control group (*p* < 0.05). Additionally, among multiparous women, those with familial sPTB had a higher number of previous sPTBs compared to the sporadic-sPTB group (*p* < 0.05). Premature newborns had a significantly lower birth weight compared to term newborns (*p* < 0.05), as expected.

### 2.1. Genetic Association Between ASTN1, EBF1, EEFSEC, MAST1, and TNF-α Gene Polymorphisms and sPTB

The genotype frequencies of *ASTN1* SNP rs146756455, *EBF1* SNPs rs2963463 and rs2946169, and *TNF-α* SNP rs1800629 were in Hardy–Weinberg equilibrium (HWE) (*p* > 0.05) across all groups. However, *MAST1* SNP rs188343966 and *EEFSEC* SNP rs201450565 were not in HWE due to a lack of variation, with minor allele frequencies (MAF) of less than 0.03 and 0.01, respectively, preventing analysis of differences in genotype and allele frequencies among the study groups.

Among tested SNPs, there was a statistically significant difference between cases and controls in the distribution of genotype for *EBF1* SNP rs2963463 and allele frequencies for *EBF1* SNP rs2946169 (Table 2). However, after applying Bonferroni correction, only *EBF1* SNP rs2963463 stayed significant (*p* adj = 0.03).

The *EBF1* SNP rs2963463 TT genotype was more prevalent in the sPTB group (8.68%) than in the control group (6.09%). Additionally, Table 3 shows that there was statistically significant difference overall between three groups (*p* = 0.015), specifically between familiar sPTB and controls (*p* = 0.017), and sporadic sPTB and controls (*p* = 0.007). However, after correction, only the difference between sporadic sPTB and controls stayed significant (*p* adj = 0.042).

For the remaining SNPs, including *ASTN1* SNP rs146756455 and *TNF-α* SNP rs1800629, no significant differences were found.

### 2.2. Genetic Association of ASTN1, EBF1, and TNF-α SNPs with Famliar sPTB vs. Sporadic sPTB vs. Controls

Table 4 shows genetic association of *ASTN1*, *EBF1*, and *TNF-α* gene polymorphisms with familiar and sporadic sPTB compared to controls, across different genetic models.

For the *ASTN1* SNP rs146756455, the CC genotype was more frequent in sporadic sPTB cases compared to controls (OR = 0.03, 95% CI = 0.00–0.57, *p* = 0.019), although this association was no longer significant after Bonferroni correction (*p* adj = 0.114).

In relation to the *EBF1* SNP rs2963463, the recessive model demonstrated a significant association between the CC genotype and a lower risk of familial sPTB compared to controls (OR = 3.91, 95% CI = 2.03–7.53, *p* adj < 0.0001) and sporadic sPTB (OR = 3.34, 95% CI = 2.40–4.64, *p* adj < 0.0001). The major T allele was also more prevalent in familial cases (OR = 2.21, 95% CI = 1.42–3.42, *p* adj < 0.0001) compared to controls. Additionally, the comparison of CC vs. TC showed a protective effect of the CC genotype in the sporadic sPTB group (OR = 3.32, 95% CI = 1.14–9.70, *p* = 0.028), but this effect lost significance after adjustment.

Moreover, the *EBF1* SNP rs2946169 minor C allele was more common in the sporadic sPTB group compared to controls (OR = 0.74, 95% CI = 0.55–0.99, *p* = 0.045), though this association was lost after adjustment.

Lastly, the *TNF-α* SNP rs1800629 minor AA genotype was more frequent in familial sPTB cases compared to both sporadic sPTB (OR = 0.29, 95% CI = 0.09–0.93, *p* = 0.036) and controls (OR = 0.25, 95% CI = 0.08–0.78, *p* = 0.018) when compared to the GG genotype. Under the recessive model, this association remained significant for both sporadic sPTB (OR = 0.29, 95% CI = 0.09–0.92, *p* = 0.038) and controls (OR = 0.26, 95% CI = 0.08–0.81, *p* = 0.020). Additionally, in terms of allele frequency, the minor allele showed significance compared to controls (OR = 0.54, 95% CI = 0.31–0.94, *p* = 0.029). After Bonferroni correction, results were no longer significant.

### 2.3. Association of ASTN1, EBF1, and TNF-α Gene Polymorphisms with Maternal and Fetal Characteristics

The correlation between maternal and fetal characteristics (gestational age at delivery, maternal age at delivery, maternal BMI, and newborn birth weight) and the genotypes of *ASTN1*, *EBF1*, and *TNF-α* was examined to explore the potential influence of these SNPs.

No significant association was found between the investigated SNPs and maternal age at delivery, maternal BMI, or newborn birth weight. However, gestational age at delivery was associated with the SNPs when sPTB was categorized into three distinct phenotypes: extremely preterm, very preterm, and moderate to late preterm. A statistically significant difference in the genotype distribution of the *ASTN1* SNP rs146756455 was observed, as the recessive CC genotype was more frequent in the extremely preterm phenotype (χ^2^ = 11.45, *p* = 0.022) (Table 5). However, after applying the Bonferroni correction, the association was no longer significant (*p* = 0.132).

In addition, we tested whether the significant association between patients and controls and the four SNPs persisted after adjustment for maternal traits that differed significantly between patients and controls according to Table 1. After adjustment for parity, none of the polymorphisms remained significant (*p* > 0.05). However, after adjusting for previous preterm birth (PTB), the *ASTN1* SNP rs146756455 became significant (χ^2^ = 4.83, *p* = 0.028), indicating a significant difference between familial and sporadic sPTB.

## 3. Discussion

In this study, we investigated the associations between polymorphisms in the candidate genes *ASTN1*, *EBF1*, *EEFSEC*, *MAST1*, and *TNF-α* and the occurrence of sPTB, revealing that the *EBF1* SNP rs2963463 may have a protective effect against sPTB in both familial and sporadic cases. This candidate gene association study represents the largest investigation of polymorphisms associated with sPTB conducted in a cohort of Caucasian women from Central Europe (Slovenia and Croatia). Examined polymorphisms of candidate genes (*ASTN1*, *EBF1*, *EEFSEC*, *MAST1*, and *TNF-α*) were selected based on previously conducted comprehensive systematic review and meta-analysis of all published genetic association studies on sPTB [8] and are examined for the first time in this population. Moreover, the stratification of the sPTB phenotype into familial and sporadic subtypes accounts for the positive family history of the included subjects, denoting this as an independent and significant risk factor for PTB.

A protective effect of *EBF1* SNP rs2963463 in both familial and sporadic sPTB was confirmed using codominant, recessive, allele, and genotype genetic models even after Bonferroni correction (*p* adj < 0.05). The analysis identified the minor CC genotype as a protective factor, reducing the risk of sPTB by 3–4 times (*p* adj < 0.05) compared to the TT+TC genotypes, with this effect evident in both familial and sporadic sPTB cases. Additionally, the major T allele of SNP rs2963463 is associated with an increased risk of sPTB in both familial and sporadic cases (*p* adj < 0.05), further positioning it as a notable risk factor. Conversely, for the *EBF1* SNP rs2946169, the minor C allele was initially associated with an increased incidence of sporadic sPTB (*p* = 0.045), suggesting that it may act as a potential risk factor. However, this association was only significant when comparing sporadic cases to controls, indicating a possible specificity to sporadic sPTB. Notably, the association lost significance after strict Bonferroni correction. These genetic findings are consistent with the largest GWAS study conducted to date, which identified *EBF1* as significantly associated with gestational duration and preterm birth [14]. Moreover, the association was subsequently confirmed as significant in a recent meta-analysis of GWAS studies by Pasanen et al. [15] and Sole-Navais et al. [16]. These results are further supported by functional studies of *EBF1*, which highlight its crucial role in regulating immune responses, cell survival, and placental development. *EBF1* influences gene expression pathways involved in immune tolerance and apoptosis, both essential for pregnancy maintenance [17]. Disruptions in these pathways, potentially due to genetic variants such as rs2963463 or rs2946169, may impair the processes needed to sustain a healthy pregnancy, thus contributing to the risk of sPTB. Recently, studies measuring *EBF1* mRNA levels during pregnancy revealed that lower mRNA expression was significantly associated with an increased risk of sPTB. Women in the lowest quartile of *EBF1* mRNA expression during the second and third trimesters had a 2.86- to 4.43-times higher risk of delivering preterm compared to those with higher mRNA levels. This suggests that polymorphic variants in the *EBF1* gene may affect mRNA expression, contributing to the risk of preterm birth [11,18]. However, the study utilized the “1879_at for *EBF1*” probe, which does not clearly correspond to a specific SNP, raising uncertainty about its precise genetic implications. When combined, these findings highlight how crucial *EBF1* is to understanding the genetic and molecular landscape of sPTB and how important it is for determining risk and possible treatment targets.

The proinflammatory *TNF-α* SNP rs1800629 variant, specifically under the allele, recessive, and codominant model, was associated with an increased risk of familiar sPTB before adjustment analysis. Minor allele A, as well as minor genotype AA, was shown to be significantly more abundant in familiar sPTB cases than both the sporadic sPTB group and controls (*p* < 0.05). Notably, the statistical difference between familial and sporadic sPTB cases suggests that distinct mechanisms may underlie these two phenotypes. In familial sPTB, the stronger significance likely points to an inherited genetic predisposition, where variants like SNP rs1800629 play a more direct and substantial role. In contrast, sporadic sPTB may involve a combination of genetic and environmental factors, which could reduce the individual impact of specific genetic variants. This highlights the possibility that familial sPTB is more heavily influenced by heritable genetic factors, while sporadic cases may result from a more complex interplay of multiple influences. To date, no other studies have specifically examined the influence of *TNF-α* in cases with a positive individual and family history of sPTB. However, several studies have reported a significant association between *TNF-α* and sPTB in sporadic cases [19,20,21,22,23], though some have failed to find a similar link [24,25,26]. In our previous meta-analysis, we confirmed a significant association between *TNF-α* and sPTB, consistent with our findings. However, the stricter Bonferroni correction in our study may explain why our results lost significance after adjustment [8]. Polymorphic variants in *TNF-α* are proven to have an effect on elevated serum levels and are often seen in women who experience sPTB [27]. Functional studies have demonstrated that the minor (A) allele of analyzed polymorphism is associated with increased *TNF-α* expression, leading to heightened inflammatory responses in the uterus and placenta. This inflammatory cascade can promote PPROM and abnormal uterine contractility, both of which contribute to the onset of preterm labor [28,29,30].

In contrast to *TNF-α*, *ASTN1* SNP rs146756455 was found to be statistically significant only in sporadic sPTB cases compared to controls under the recessive model (*p* < 0.05), suggesting that the minor CC genotype may act as a risk factor for sPTB. Additionally, when comparing *ASTN1* genotypes and allele frequencies across gestational age phenotypes, the minor CC genotype was more frequently observed in extremely early sPTB cases (<28 weeks) (Table 5). However, after applying the Bonferroni correction, both associations lost statistical significance, which could partly be due to the fact that 76% of our cases had moderate to late sPTB. *ASTN1* functions as a glycoprotein crucial for glial-guided neuronal migration during brain development [31]. Gupta et al.’s GWAS study identified an association between *ASTN1* and sPTB, suggesting that it may have broader roles beyond neuronal migration [12]. Its involvement in cell adhesion and migration might extend to similar processes in placental or uterine tissues during pregnancy. Further research is required to better understand the genetic mechanisms through which *ASTN1* contributes to preterm labor.

Additionally, SNPs in the *EEFSEC* and *MAST1* genes were examined, but differences could not be determined as all individuals had the same genotype, with the remaining genotypes being extremely rare. Detecting these rare genotypes would require a substantially larger sample population to identify homozygous recessive individuals. Specifically, the MAF for *EEFSEC* is less than 0.01, and for *MAST1* it is less than 0.03, which could explain the limited variation observed between tested groups of less than 300. For instance, *EEFSEC* was initially identified in a cohort of 43,568 women, with an additional 8643 participants in the replication study [14]. Alternatively, this SNP may be associated with a more severe phenotype, such as in the original study where the inclusion criteria required a history of PTB before 34 weeks [32].

In terms of maternal characteristics, the recessive CC genotype of the *ASTN1* SNP rs146756455 was more frequently observed in mothers who experienced extremely early preterm birth, delivering before 28 weeks of gestation. This suggests that the CC genotype may be associated with more severe cases of PTB. Adjusting for previous sPTB in the same gene highlighted a previously hidden relationship between *ASTN1* and the sPTB subgroups, suggesting that this polymorphism may be more closely linked to cases involving a history of preterm birth, particularly familial ones. This raises the question of whether other maternal and fetal characteristics may reveal hidden genetic associations when carefully adjusted for.

Future studies should aim to further explore the genetic mechanisms underlying sPTB by focusing on cohorts with an earlier onset of sPTB and a positive family history on the maternal side. The inclusion of a higher prevalence of patients with moderate to late preterm birth (gestational age 32–37 weeks) may limit the discovery of genetic risk factors involved in the pathogenesis of sPTB. Additionally, larger study populations are essential to improve the detection of SNPs with lower minor allele frequencies and to enhance the statistical power required to identify significant associations with rare variants. Incorporating advanced genomic approaches, such as whole genome sequencing and epigenomic profiling, could provide deeper insights into gene–gene and gene–environment interactions. Functional studies, particularly those investigating SNPs, particularly rs2963463 in *EBF1* gene, which passed the stringent Bonferroni correction, are needed to elucidate the biological processes contributing to both familial and sporadic sPTB. This comprehensive approach may help in identifying potential biomarkers and therapeutic targets for recognizing, and in an ideal scenario preventing, preterm birth across various populations.

## 4. Materials and Methods

### 4.1. Patients

This case-control study enrolled women who gave birth at the Department of Obstetrics and Gynecology of the Clinical Hospital Center of Rijeka and Osijek, Croatia and the University Medical Center in Ljubljana, Slovenia from 2018 to 2023. During this period, eligible women were invited to participate in the study, and those who agreed provided written informed consent prior to enrollment. The study was approved by the Ethics Committee for Biomedical Research of the Faculty of Medicine of the University of Rijeka (2170-29-02/1-19-2) and the University of Osijek (602-04/18-08/07) and by the Slovenian National Medical Ethics Committee (98/12/10).

A total of 292 women with sPTB (179 Croatian and 113 Slovenian) and 281 control women (157 Croatian and 124 Slovenian) were included in the study. The demographic and clinical data of the women with sPTB and their newborns were collected according to the guidelines for genetic epidemiology studies of PTB using a self-developed, interviewer-administered questionnaire, as described in our previous studies [33,34]. The study population, consisting of women from Croatia and Slovenia, represents a homogeneous population of Central and Southeast European ancestry.

The patient group (sPTB group) was divided into two subgroups: 44 women with a positive personal and family history in whom a first-degree maternal relative (mother or sister) had sPTB before 37 weeks’ gestation (*familial sPTB group*), and 248 women with a positive personal but negative family history of sPTB before 37 weeks’ gestation (*sporadic sPTB group*). All women with sPTB had a singleton pregnancy after natural conception with spontaneous onset of PTB before 37 weeks’ gestation. Gestational age was determined based on the last menstrual period and confirmed by ultrasonography in the first trimester. In cases where the estimated gestational age from the last menstrual period and the ultrasound examination differed by more than 7 days, the gestational age was adjust-ed according to the ultrasound measurement in the first trimester [35]. Strict exclusion criteria for all known iatrogenic and disease factors (i.e., in vitro fertilization, infections of the birth canal, diabetes, hypertension, renal disease, autoimmune disease, and pregnancy complications) were applied to focus exclusively on sPTB. In addition, none of the live-born children showed congenital anomalies or signs of infection.

The control group consisted of 283 healthy women who had at least one full-term singleton birth (38–42 weeks of gestation) following an uncomplicated pregnancy, resulting in a healthy child without congenital anomalies.

### 4.2. DNA Isolation and Genotyping

Genomic DNA was isolated from peripheral blood leukocytes using the Qiagen FlexiGene DNA Kit (Qiagen GmbH, Hilden, Germany) and its quality and concentration measured using a NanoDrop spectrophotometer (Thermo Fisher Scientific, Waltham, MA, USA).

The SNPs *ASTN1* rs146756455, *EBF1* rs2963463, *EBF1* rs2946169, *EEFSEC* rs201450565, *MAST1* rs188343966, and *TNF-α* rs1800629 were analyzed using TaqMan Pre-designed and Custom SNP Genotyping Assays (Applied Biosystems, Foster City, CA, USA) on a StepOne Real-Time PCR System (Applied Biosystems, Foster City, CA, USA) according to the manufacturer’s recommendations. PCR cycling conditions were adjusted according to the manufacturer’s recommendations.

### 4.3. Statistical Anlysis

Statistical analysis was performed using Statistica for Windows, ver. 14.0 (StatSoft, Inc., Tulsa, OK, USA). For all tests performed, the statistical significance level was 0.05. The calculations of statistical significance were performed using the GAS Power Calculator: https://csg.sph.umich.edu/abecasis/cats/gas_power_calculator/ (accessed on 1 September 2024).

To assess significant differences in genotype, allele, and haplotype frequencies between groups, the Pearson chi-square (χ^2^) test was used for categorical variables. ANOVA or the Kruskal–Wallis test was applied for continuous variables, depending on the normality of the distribution. Normality of distribution was tested with the Kolmogorov–Smirnov test. Odds ratios with 95% confidence intervals were estimated as a measure of genetic association with sPTB. Four comparative models were used: allelic contrast, dominant, recessive, and codominant. To examine the association between birth weight, gestational week, and BMI, Kruskal–Wallis test with a factorial design was used, followed by the Scheffé post hoc test. For maternal age and for smoking, previous PTB, and parity, ANOVA with Tukey post hoc test and Pearson chi-square test were employed, respectively. Control of the effect of confounding variables was performed by multiple regression analysis. To account for the risk of false positives when performing multiple comparisons, Bonferroni correction was applied. The level of statistical significance was set at *p* < 0.05.

## 5. Conclusions

Our findings indicate that the *EBF1* SNP rs2963463, which meets the stringent Bonferroni correction threshold, acts as a significant protective factor in both familial and sporadic sPTB. Specifically, mothers carrying the minor CC genotype of rs2963463 have a 3–4-times lower risk of sPTB, highlighting its protective role. In contrast, three potential risk factors emerged: the *TNF-α* SNP rs1800629 polymorphism was more prevalent in familial cases compared to sporadic cases and controls, while *EBF1* SNP rs2946169 and *ASTN1* SNP rs146756455 was more common in sporadic cases compared to controls. This suggests that different underlying mechanisms may contribute to the familial and sporadic forms of sPTB.

## Figures and Tables

**Table 1:** Characteristics of women with familiar and sporadic sPTB and controls.

	Cases (N = 292)		Controls (N = 281)	*p*
	Familiar sPTB (N = 44)	Sporadic sPTB (N = 248)		
**Maternal characteristics**				
**Mean age at delivery/median (range)**	31 (22–40)	31 (16–44)	30 (19–43)	0.128 ^1^
**Prepregnancy BMI/median (range)**	23 (17–32)	25 (19–39)	24 (16–39)	0.202 ^2^
**Gestational age at delivery/median (range)**	34 (24–36)	35 (21–36)	40 (37–41)	**0.000** ^2^
Extremely preterm < 28 weeks/N (%)	5 (11.4)	20 (8.3)		
Very preterm 32–28 weeks/N (%)	6 (13.6)	37 (15.2)		
Moderate to late preterm 32–36 weeks/N (%)	33 (75.0)	186 (76.5)		
**Smoking during pregnancy** Yes/N (%) No/N (%)	6 (13.6) 38 (86.4)	46 (18.9) 197 (81.1)	40 (18.4) 177 (81.6)	0.702 ^3^
**Parity** Nulliparous/N (%) Multiparous/N (%)	15 (35.7) 27 (64.3)	46 (20.1) 183 (79.9)	33 (15.2) 184 (84.8)	**0.008** ^3^
**Previous sPTB** Yes/N (%) No/N (%)	11 (25.0) 33 (75.0)	24 (9.9) 218 (90.1)		**0.005** ^3^
**Fetal characteristics**				
Birth weight (grams)/median (range)	2170 (650–3400)	2269 (576–3550)	3460 (2380–4740)	**0.000** ^2^

Bold denotes statistical significance; epidemiological data about *smoking during pregnancy* and *parity* were available for familiar 42/44 (95.5%) and 229/248 (92.3%) sporadic sPTB, and 217/281 (77.2%) controls; data about *previous sPTB* were available for woman with familiar 44/44 (100.0%) and 242/248 (97.6%) sporadic sPTB. ^1^ One-way ANOVA. ^2^ Kruskal–Wallis. ^3^ χ^2^ test.

**Table 2:** Genotype and allele frequencies of *ASTN1*, *EBF1*, *EEFSEC*, *MAST1*, and *TNF-α* gene polymorphisms among cases and controls.

		Cases (N = 292)	Controls (N = 281)	χ^2^ *	*p*	*p* adj **
***ASTN1* rs146756455 G/C**						
genotype	GG	277 (95.85)	264 (95.31)	1.23	0.542	3.276
	GC	11 (3.81)	13 (4.69)			
	AC	1 (0.34)	0			
allele	G	564 (97.58)	538 (97.11)	0.24	0.626	2.136
	A	14 (2.42)	16 (2.89)			
***EBF1* SNP rs2963463 T/C**						
genotype	TT	25 (8.68)	17 (6.09)	10.47	**0.005**	**0.030**
	TC	156 (54.1)	121 (43.37)			
	CC	107 (37.22)	141 (50.54)			
allele	T	182 (31.60)	155 (27.78)	1.98	0.160	0.960
	C	394 (68.40)	403 (72.22)			
***EBF1* rs2946169 C/T**						
genotype	CC	165 (57.10)	180 (64.52)	4.78	0.092	0.552
	CT	103 (35.64)	88 (31.54)			
	TT	21 (7.26)	11 (3.94)			
allele	C	433 (74.91)	450 (80.65)	5.38	**0.020**	0.120
	T	145 (25.09)	108 (19.35)			
***TNF-α* rs1800629 G/A**						
genotype	GG	208 (71.23)	213 (75.80)	7.68	0.104	0.624
	GA	70 (23.97)	59 (21.00)			
	AA	14 (4.80)	9 (3.20)			
allele	G	486 (83.22)	485 (86.47)	2.10	0.148	0.888
	A	98 (16.78)	77 (13.70)			

Bold denotes statistical significance. * Pearson chi-square test. ** Bonferroni correction. Genotype data were available for *ASTN1* cases 289/292 (99.0%) and 277/281 (98.6%) controls; *EBF1* cases 288/292 (98.6%) and 279/281 (93.3%) controls; and *TNF-α* cases 292/292 (100.0%) and 281/281 (100.0%) controls.

**Table 3:** Genetic association analysis of *EBF1* SNP rs2963463 across familial sPTB, sporadic sPTB, and controls, using different inheritance/genetic models.

		Cases		Controls (N = 281)	χ^2^	*p*	*p* adj *
		Familiar sPTB (N = 44)	Sporadic sPTB (N = 248)				
***EBF1* SNP rs2963463 T/C**							
genotype	TT	6 (13.64)	19 (7.79)	17 (6.09)	12.34	**0.015 ^a^**	0.300
	TC	23 (52.27)	133 (54.51)	121 (43.37)			
	CC	15 (34.09)	92 (37.70)	141 (50.54)			
allele	T	35 (39.77)	147 (30.12)	155 (27.78)	10.85	0.210	1.260
	C	53 (60.23)	341 (69.88)	403 (72.22)			

Bold denotes statistical significance. * Bonferroni correction. ^a^ Familial sPTB vs. controls χ^2^ = 5.69, *p* = 0.017, *p* adj = 0.102; sporadic sPTB vs. controls χ^2^ = 7.37, *p* = 0.007, *p* adj = 0.042.

**Table 4:** Genetic association analysis of *ASTN1*, *EBF1*, and *TNF-α* SNPs using different inheritance/genetic models.

Genetic Models	Familial sPTB vs. Sporadic sPTB			Familial sPTB vs. Controls			Sporadic sPTB vs. Controls		
	OR (95% CI)	*p*	*p* adj *	OR (95% CI)	*p*	*p* adj *	OR (95% CI)	*p*	*p* adj *
***ASTN1* rs146756455**									
GG vs. GC+CC	4.76 (0.28–81.95)	0.282	1.692	4.54 (0.27–77.79)	0.296	1.776	0.96 (0.43–2.14)	0.913	5.478
GG+GC vs. CC	0.55 (0.02–13.62)	0.712	4.272	0.16 (0.00–8.19)	0.362	2.172	0.03 (0.00–0.57)	**0.019**	0.114
GG vs. GC	4.38 (0.25–75.74)	0.309	1.854	4.54 (0.27–77.79)	0.296	1.776	1.04 (0.46–2.37)	0.920	5.520
GG vs. CC	0.57 (0.00–14.26)	0.733	4.398	0.17 (0.00–8.89)	0.374	2.244	0.29 (0.01–7.26)	0.455	2.730
CC vs. GC	7.67 (0.11–550.17)	0.350	2.100	27.00 (0.22–3382.54)	0.181	1.086	3.52 (0.13–95.09)	0.454	2.724
G vs. C	5.39 (0.32–91.12)	0.243	1.458	5.42 (0.32–91.22)	0.240	1.440	1.01 (0.49–2.09)	0.976	5.856
***EBF1* SNP rs2963463**									
TT vs. TC+CC	1.87 (0.70–4.98)	0.211	1.266	2.43 (0.90–6.56)	0.079	0.474	1.30 (0.66–2.56)	0.446	2.676
TT+TC vs. CC	1.170 (0.60–2.30)	0.648	3.888	3.91 (2.03–7.53)	**0.000**	**0.000**	3.34(2.40–4.64)	**0.000**	**0.000**
TT vs. TC	1.83 (0.66–5.06)	0.247	1.482	1.86 (0.66–5.21)	0.240	1.440	0.98 (0.49–1.98)	0.963	5.778
TT vs. CC	1.94 (0.67–5.63)	0.225	1.350	3.32 (1.14–9.70)	**0.028**	0.168	1.71 (0.85–3.47)	0.135	0.810
CC vs. TC	0.94 (0.47–1.90)	0.870	5.220	0.56 (0.28–1.12)	0.101	0.606	0.594 (0.41–0.85)	**0.005**	**0.030**
T vs. C	1.57 (1.02–2.44)	**0.043**	0.258	2.21(1.42–3.42)	**0.000**	**0.000**	1.40 (1.08–1.82)	**0.012**	0.072
***EBF1* rs2946169**									
CC vs. CT+TT	0.71 (0.37–1.58)	0.303	1.818	0.55 (0.29–1.04)	0.067	0.402	0.77 (0.54–1.10)	0.149	0.894
CC+CT vs. TT	1.08 (0.31–3.85)	0.901	5.406	0.56 (0.15–2.10)	0.390	2.340	0.52 (0.24–1.12)	0.094	0.564
CC vs. CT	0.68 (0.35–1.33)	0.260	1.560	0.57 (0.29–1.10)	0.093	0.558	0.83 (0.57–1.21)	0.332	1.992
CC vs. TT	0.92 (0.25–3.39)	0.904	5.424	0.45 (0.12–1.73)	0.244	1.464	0.49 (0.22–1.06)	0.070	0.420
TT vs. CT	1.36 (0.36–5.08)	0.650	3.900	0.79 (0.20–3.11)	0.738	4.428	0.58 (0.26–1.31)	0.191	1.146
C vs. T	0.82 (0.49–1.36)	0.435	2.610	0.60 (0.36–1.01)	0.052	0.312	0.74 (0.55–0.99)	**0.045**	0.270
***TNF-α* rs1800629**									
GG vs. GA+AA	0.75 (0.38–1.47)	0.398	2.388	0.62 (0.31–1.22)	0.165	0.990	0.83 (0.56- 1.22)	0.343	2.058
GG+GA vs. AA	0.29 (0.09–0.92)	**0.036**	0.216	0.26 (0.08–0.81)	**0.020**	0.120	0.88 (0.34- 2.25)	0.787	4.722
GG vs. GA	0.97 (0.45- 2.11)	0.943	5.658	0.80 (0.37–1.74)	0.579	3.474	0.83 (0.55- 1.25)	0.363	2.178
GG vs. AA	0.29 (0.09–0.93)	**0.038**	0.228	0.25 (0.08–0.78)	**0.018**	0.108	0.84 (0.33- 2.16)	0.718	4.308
AA vs. GA	3.33 (0.93- 12.01)	0.066	0.396	3.28 (0.91–11.82)	0.070	0.420	0.98 (0.36–2.65)	0.974	5.844
G vs. A	0.63 (0.36–1.10)	0.108	0.648	0.54 (0.31–0.94)	**0.029**	0.174	0.85 (0.61–1.20)	0.353	2.118

Bold denotes statistical significance. * Bonferroni correction.

**Table 5:** Genetic association analysis of selected SNPs in *ASTN1*, *EBF1*, and *TNF-α* genes among three phenotypes of sPTB categorized by gestational age at delivery.

		Extremely Preterm (<28 Weeks) (N = 26)	Very Preterm (32–28 Weeks) (N = 42)	Moderate to Late Preterm (32–37 Weeks) (N = 217)	χ^2^ *	*p*	*p* adj **
***ASTN1* rs146756455**							
genotype	GG	24 (8.79)	39 (14.29)	210 (76.92)	11.45	**0.022**	0.132
	GC	1 (9.09)	3 (27.27)	7 (63.64)			
	CC	1 (100)	0 (0)	0 (0)			
allele	G	49 (94.23)	81 (96.43)	427 (98.39)	4.34	0.114	0.684
	C	3 (5.77)	3 (3.57)	7 (1.61)			
***EBF1* SNP rs2963463**							
genotype	TT	3 (12)	3 (12)	19 (76)	0.78	0.942	5.652
	TC	13 (8.55)	22 (14.47)	117 (77.63)			
	CC	10 (9.35)	18 (16.82)	79 (73.83)			
allele	T	19 (36.54)	28 (32.56)	155 (36.05)	0.40	0.817	4.902
	C	33 (63.46)	58 (67.44)	275 (63.95)			
***EBF1* rs2946169**							
genotype	CC	15 (9.20)	26 (15.95)	122 (74.85)	0.25	0.993	5.958
	CT	9 (8.91)	14 (13.86)	78 (77.23)			
	TT	2 (9.52)	3 (14.29)	16 (76.19)			
allele	C	39 (75.00)	66 (76.74)	322 (74.54)	0.19	0.911	5.466
	T	13 (25.00)	20 (23.26)	110 (25.46)			
***TNF-α* rs1800629**							
genotype	GG	17 (8.33)	33 (16.18)	154 (75.49)	1.62	0.080	0.480
	GA	7 (10.00)	9 (12.86)	54 (77.14)			
	AA	2 (14.29)	1 (7.14)	11 (78.57)			
allele	G	41 (78.85)	75 (87.21)	362 (82.65)	1.75	0.416	2.496
	A	11 (21.15)	11 (12.79)	76 (17.35)			

Bold denotes statistical significance. * Pearson chi-square test. ** Bonferroni correction.

## Data Availability

The datasets generated and analyzed as part of the current study are not publicly accessible, as the patients in this study have given informed consent, which does not allow the data to be published in public databases. However, the data are available upon reasonable request. Requests should be addressed to corresponding author.
